# Mucosal Microbiota from Colorectal Cancer, Adenoma and Normal Epithelium Reveals the Imprint of *Fusobacterium nucleatum* in Cancerogenesis

**DOI:** 10.3390/microorganisms11051147

**Published:** 2023-04-28

**Authors:** Orazio Palmieri, Stefano Castellana, Anna Latiano, Tiziana Latiano, Annamaria Gentile, Anna Panza, Marianna Nardella, Davide Ciardiello, Tiziana Pia Latiano, Giuseppe Corritore, Tommaso Mazza, Francesco Perri, Giuseppe Biscaglia

**Affiliations:** 1 Division of Gastroenterology and Endoscopy, Fondazione IRCCS “Casa Sollievo della Sofferenza”, 71013 San Giovanni Rotondo, Italy; 2 Unit of Bioinformatics, Fondazione IRCCS “Casa Sollievo della Sofferenza”, 71013 San Giovanni Rotondo, Italy; 3 Division of Oncology, Fondazione IRCCS “Casa Sollievo della Sofferenza”, 71013 San Giovanni Rotondo, Italy; 4 Division of Medical Oncology, Department of Precision Medicine, University of Campania Luigi Vanvitelli, 80131 Naples, Italy

**Keywords:** colorectal cancer, adenomas, microbiota, *Fusobacterium nucleatum*

## Abstract

An increasing amount of evidence suggests the emerging role of the gut microbiota in the development of colorectal cancer (CRC). This study aimed to elucidate the architecture of microbial communities within normal and neoplastic colonic mucosa. Methods: Microbiota were analyzed by NGS and by an ensemble of metagenomics analysis tools in a total of 69 tissues from 9 patients with synchronous colorectal neoplasia and adenomas (27 specimens: 9 from normal tissues, 9 adenomas, and 9 tumours), 16 patients with only colonic adenomas (32 specimens: 16 from normal tissues and 16 adenomas), and from healthy subjects (10 specimens of normal mucosa). Results: Weak differences were observed in alpha and beta metrics among the synchronous tissues from CRC and controls. Through pairwise differential abundance analyses of sample groups, an increasing trend of *Rikenellaceae, Pseudomonas* and *Fusobacterium,* and decreasing trends of *Staphylococcus*, *Actinobacillus* and *Gemmiger* were observed in CRC, while *Staphylococcus* and *Bifidobacterium* were decreased in patients with only adenomas. At RT-qPCR analysis, *Fusobacterium nucleatum* was significantly enriched in all the tissues of subjects with synchronous colorectal neoplasia. Conclusion: Our findings provide a comprehensive view of the human mucosa-associated gut microbiota, emphasizing global microbial diversity mostly in synchronous lesions and proving the constant presence of *Fusobacterium nucleatum,* with its ability to drive carcinogenesis.

## 1. Introduction

Colorectal cancer (CRC) significantly contributes to the global health burden. In 2022, approximately 1.9 million new cases and 0.9 million deaths worldwide were reported [1]. It is well known that carcinogenesis in the large bowel has a multifactorial etiology that includes genetic, environmental and lifestyle factors [2].

Among the genetic factors, hereditary cancer syndromes, such as Lynch syndrome (LS), or familial adenomatous polyposis (FAP), are implicated in 5–10% of CRC cases [3]. Approximately 25% of CRC cases have a family history of CRC with infrequent or unclear genetic markers, while the remaining cases arise sporadically [3].

The relatively high level of sporadic CRC reflects the importance of environmental factors, both exogenous and endogenous, that may contribute to carcinogenesis. It has been hypothesized that one of the players is the microbial community living in the large bowel. In this milieu, the microbiota or the metabolites generated through microbial processes have a profound effect on immune cells and their functional states [4], and could be proximate environmental modifiers of risk for CRC.

To date, the use of biomarkers has been limited. Universal screening for mismatch repair proteins has been proposed to identify potential individuals with Lynch syndrome [5]. Metagenomic studies performed in the stool or in mucosal tissues revealed microbial composition and ecological changes, providing evidence for the correlation between a dysbiotic microbial community and CRC [6]. The fecal microbiota only partially reflect the mucosal microbiota [7].

Animal models allowed the identification of specific microbes associated with colonic carcinogenesis. Among them are strains of *Escherichia coli* [8], *Bacteroides fragilis* [9] and *Fusobacterium nucleatum* [10]. Among the microbes identified in the numerous studies of patients with CRC, *Fusobacterium nucleatum* has emerged as strongly associated with pathogenesis, progression, and treatment response in CRC [11].

To highlight the impact of the overall architecture of microbial composition on the sequence adenoma–carcinoma, we imagined a model represented by subjects with coexisting neoplastic tissue, adenoma tissue and normal tissue, and those with only adenomas.

Therefore, we analyzed the 16S ribosomal RNA (rRNA) microbiota by next generation sequencing (NGS) from two groups of patients: subjects with synchronous carcinoma and adenoma (biopsy pinched on neoplasms, on adenoma and adjacent normal tissue) and subjects with only adenoma (biopsy pinched on adenoma and adjacent normal tissue). In addition, using RT-qPCR, we focused on *Fusobacterium nucleatum*, known as a mutualist, infectious agent, and oncogenic microorganism [12].

## 2. Materials and Methods

### 2.1. Clinical Samples

A total of 69 biopsies from 25 patients and 10 healthy controls were prospectively assessed at the Gastroenterology and Digestive Endoscopy Unit at Fondazione IRCCS-Casa Sollievo della Sofferenza Hospital. Of these, 9/25 (36%) were CRC patients with synchronous adenomas, while the remaining 16 had adenomas without neoplasia. For all the patients the normal tissues were also collected. The specimens were collected during the endoscopic examination and were immediately frozen in liquid nitrogen and stored at −80 °C. Biopsy samples from 10 healthy controls were also collected. No inflammatory polyps, hyperplastic polyps, non-advanced adenomas, or other intestinal diseases were found during the colon examinations.

The study was conducted following the Declaration of Helsinki, and the protocol was approved by the Hospital’s Ethics Committee (Prot. N.132 CE/2015). Informed consent was signed by all subjects. To limit the impact of genetics and drugs, subjects having an antecedent diagnosis of inherited cancer syndrome, an age of CRC diagnosis under 50 years, and treatment with antibiotics and/or chemotherapy within the past three months were excluded. In addition, no dietary habits and overweight/obesity presence were recorded.

### 2.2. Laboratory Procedures

DNA was extracted using the AllPrep Power viral DNA/RNA Kit (Qiagen, Hilden, Germany), following the manufacturer’s recommendations. The gentleMACS Dissociator (Miltenyi Biotec, Bergisch-Gladbach, Germany) was used for the homogenization step of the tissue samples. DNA quantity was examined using a NanoDrop ND-1000 spectrophotometer (Thermo Fisher Scientific, Inc. Somerset, NJ, USA). Microbial diversity in the mucosal specimens was studied by sequencing the amplified V3 to V4 hypervariable region of the 16S rRNA gene. PCR primers and conditions followed the Illumina 16S Metagenomic Sequencing Library preparation guide (Part # 15.044.223 Rev.B) [13] with the following exceptions: for the first 16S PCR the process was performed using Taq Phusion High-Fidelity (Thermo Fisher Scientific, Sunnyvale, CA, USA) in 25 µL reaction volumes, and 25 cycles were used in the PCR. A PCR negative control, consisting of PCR-grade water, was systematically added to each PCR run. Amplicons were detected using 2% agarose gel electrophoresis using 1 µg/mL ethidium bromide in the presence of a 1 kb ladder as a marker in the first line.

Subsequently, amplicons of about 550 bp were considered positive and were purified using AMPure XP beads (Beckman Coulter, Milan, Italy). Afterwards, the ligation of the dual indexing adapters was performed in the presence of Nextera XT Index Primer 1 and Primer 2 (Illumina, San Diego, CA, USA), Taq Phusion High-Fidelity (Thermo Fisher Scientific), and 5 μL purified DNA, according to the manufacturer’s instructions. The products were purified using AMPure XP beads to create the final DNA library. The libraries’ concentration and fragment size were measured using a fluorometric-based system (Qubit dsDNA BR Assay System; Thermo Fisher Scientific) and the Agilent 2200 TapeStation Bioanalyzer (HS D1000 ScreenTape Assays; Agilent Technologies, Santa Clara, CA, USA), respectively. Equal amounts of DNA libraries were pooled, denatured with NaOH, diluted with hybridization buffer to 7 pM following the Illumina protocol, and spiked with 20% PhiX (Illumina). The libraries were loaded into a flow cell V2 (500 cycles) by paired-end sequencing (2 × 250) (Illumina) and sequenced with MiSeq sequencing instrument System (Illumina) according to the manufacturer’s recommendations.

### 2.3. Quantitative Real-Time PCR (RT-qPCR)

The RT-qPCR experiment was performed using the Microbial DNA qPCR Assay Kit (Qiagen) which is based on the detection of bacterial species targeting the 16s rRNA gene conserved across a wide variety of microorganisms. An aliquot (0.3 μL) of mucosal DNA dilution was used in a 10 μL reaction mixture, containing 5 μL of Microbial qPCR Mastermix and 0.4 μL Microbial DNA qPCR Assay. For each sample, different reactions were carried out: a *Fusobacterium nucleatum* assay (BPID00160A), a Positive PCR Control (PPC) reaction, and a Pan Bacteria 1 reaction. The cycling conditions were: denaturation (95 °C for 10 min) followed by 40 cycles of amplification (95 °C for 15 s and 60 °C for 2 min) using ABI 7900 HT (ThermoFisher Scientific). The results were evaluated by SDS version 2.4 software and qPCR data were analyzed using the 2∧−(delta Ct) method.

### 2.4. Bioinformatics and Statistical Data Analysis

The quality of sequencing output was primarily inspected by FastQC application; then, sample reads were demultiplexed and analyzed using the QIIME2 v.2021.11 suite [14]. The initial steps, which included read quality checking and filtering, de-replication, chimeric reads identification, paired-end reads joining, and sequence clustering, were performed using the DADA2 [15] plugin and a raw feature table was generated. The taxonomic classification for the detected features was obtained using the QIIME2 embedded Naïve Bayes fitted classifier, pre-trained on the most recent Greengenes reference database (ver. 13.8) [16]. Rarefaction curve analysis using the QIIME2 diversity plugin was used to estimate the completeness of microbial communities’ sampling. Subsequently, alpha indexes (Shannon’s diversity index [17], number of observed OTUs, Faith’s Phylogenetic Diversity [18], Pielou’s evenness [19]) and beta dissimilarities (Jaccard distance [20], Bray–Curtis distance [21], unweighted UniFrac and weighted UniFrac distances [22]) were calculated. Jaccard and unweighted UniFrac dissimilarity are “qualitative” as they are based on the presence/absence of taxonomic features (Jaccard) or the fraction of unshared ones (along phylogenetic tree) across samples (Unifrac). Bray–Curtis and weighted Unifrac operate through quantities, i.e., take into account counts of shared features (Bray–Curtis) or weights for unshared “unique” features. These latter distances are more sensitive, although, in standard practice, both qualitative and quantitative measures are calculated and evaluated.

Principal Coordinates Analysis (“PCoA”) plots using the EMPeror application [23] for each of the beta diversity metrics were generated to check sample/experimental heterogeneity. Kruskal–Wallis and PERMANOVA statistical tests were applied to detect differences in the above-mentioned alpha index and beta measures among sample groups, respectively.

Variation in the taxa abundance profiles among groups was explored by compositional data analysis methods, i.e., QIIME2-ANCOM, coda-lasso, clr-lasso and Selbal [24]. Input for QIIME2-ANCOM consisted of two filtered feature tables, collapsed, respectively, at the species and genus levels. We removed contaminant sequences (i.e., of mitochondrial/chloroplast origin) and “ultra-rare” features, appearing in less than 5 samples (i.e., around the 10% of the total considered samples, 53) or with less than 20 counts (across all samples).

Coda-lasso [25], clr-lasso [26] and Selbal feature selection algorithms were additionally implemented (as described in https://malucalle.github.io/Microbiome-Variable-Selection/, accessed on 4 October 2022) in order to retrieve a sort of microbial signature in pairwise group comparisons (Normal_P_T, Polyps_P_T, Tumour, Normal_P, Polyps_P, respectively, from synchronous/lesions_PT and Adenomas_P_T, and from Healthy Controls_HC).

Furthermore, the genetic and functional content within samples was predicted by using the Picrust v2.3.0_b software [27], using the QIIME2 filtered feature table and “representative sequences (rep-seqs.qza file)” as input data. Picrust2 predicted counts for pathways (namely, “unstratified pathway abundance” count table, with pathways represented by their relative MetaCyc [28] were further analyzed through the compositionally aware tool, ALDEx. Analogously to the binary comparisons undertaken for microbial signature analysis, the global pathway table was basically split according to the sample groups, and the “aldex” command for pairwise analysis was run. From the raw output, three ALDEX2 “effect” cutoffs of “1”, “1.5” and “2” (absolute value, from relaxed to stringent) were chosen to evidence “differentially abundant” pathways within each comparison. Statistical and graphics analyses were carried out within the R statistical environment, through the packages Microbiome v1.8.0 [29], ggplot2 v3.3.3 [30] and Phyloseq v.1.30.0 [31].

## 3. Results

### 3.1. Clinicopathologic Characteristics and Diversity Analysis

A total of 69 mucosal biopsy specimens were collected. Of them, 26 specimens were from patients with CRC and synchronous adenomatous polyps; biopsies were pinched from colorectal neoplasia (Tumour; N = 9), adenomas (Polyps_P_T; N = 8) and adjacent normal tissues (Normal_P_T; N = 9). All adenomas had the same localization of cancers (distal or proximal to splenic flexure). Features of advanced adenoma (polyp greater than 10 mm, villous histology, a severe dysplasia) [32] were identified. When more than one polyp was observed, the most advanced one was considered. DNA extracted from one adenoma specimen, namely, SGR5P, returned no PCR, and was excluded from any further analysis. Other 32 specimens were pinched from adenomatous polyps (Polyps_P; N = 16) and adjacent normal tissues (Normal_P; N = 16). The general characteristics of the participants are given in Table 1. The control group consisted of 10 healthy non-CRC and non-hospitalized individuals (Normal_HC) with normal colonic mucosa (5 females; mean age at recruitment 47.1 ± 16.2; 6 from left or distal colon, 4 from right or transverse colon).

### 3.2. 16 S rRNA V3-V4 Region Sequencing

After the QIIME2 Quality Control procedure, we obtained a sample-specific sequencing yield ranging from 6417 to 73,116 good-quality reads. Given the raw feature table and the corresponding phylogenetic tree, we ran several rarefaction tests with different read depth cutoffs (see Supplementary File S1) to establish an optimal sampling depth with a minimal sample loss. Rarefaction at a depth of 9103 reads determined the exclusion of only two poorly sequenced samples (one patient with adenomas and one with synchronous tissues) while guaranteeing the stable distribution of three alpha diversity metrics in all the investigated groups.

### 3.3. Microbial Diversity and Community Analyses

Some weak differences in alpha and beta diversities emerged between healthy controls and patients. In detail, alpha diversity was evaluated by analyzing four metrics (Shannon Entropy, Pielou’s evenness, number of observed features, and Faith’s Phylogenetic Distance) and comparing group-specific distributions through Kruskal–Wallis tests, globally and pairwise. In Table 2 are reported the *q*-values for pairwise group comparisons, while boxplots for diversity analyses are shown in Supplementary File S1.

When normal tissues or polyps or tumors from synchronous lesions (HC vs. Normal_P_T, HC vs. Polyps_P_T and HC vs. Tumor, respectively) were compared with HC, we highlighted that the microbial communities were characterized by a lower diversity, although no significant differences emerged at all the analyzed alpha index values (*q*-values > 0.05).

We also compared four beta dissimilarity metrics (Bray–Curtis, Jaccard, Unweighted Unifrac, and Weighted Unifrac) through the PERMANOVA pairwise test and visualized sample dissimilarity through Emperor Plots (Supplementary File S1).

Comparison between tissues from synchronous samples and HC showed a statistically significant difference in terms of Jaccard dissimilarity for all the analyzed groups (*q*-value < 0.05). The difference was significant also for Bray–Curtis dissimilarity at the tumor level (*q*-value = 0.03), while it was weakly associated to Normal_P_T (*q*-value = 0.05).

When we compared the microbiota of HC to those with adenomas and adjacent normal tissues from the same subject, the differences among the samples was significantly different only for Jaccard dissimilarity (Normal_P, *q*-value = 0.026; Polyps_P, *q*-value = 0.015) (Table 3).

For the “paired” samples, i.e., Normal_P_T, Polyps_P_T and Tumor, given the limited sample size, no significant differences in terms of alpha and beta diversity indices were found. Pairwise Kruskal–Wallis tests for Shannon Entropy, evenness, number of observed OTUs and Faith’s Phylogenetic Distance group distributions gave no significant results. The distance among such groups (PERMANOVA tests for four beta diversity measures) was also not significant.

**Figure 1:**
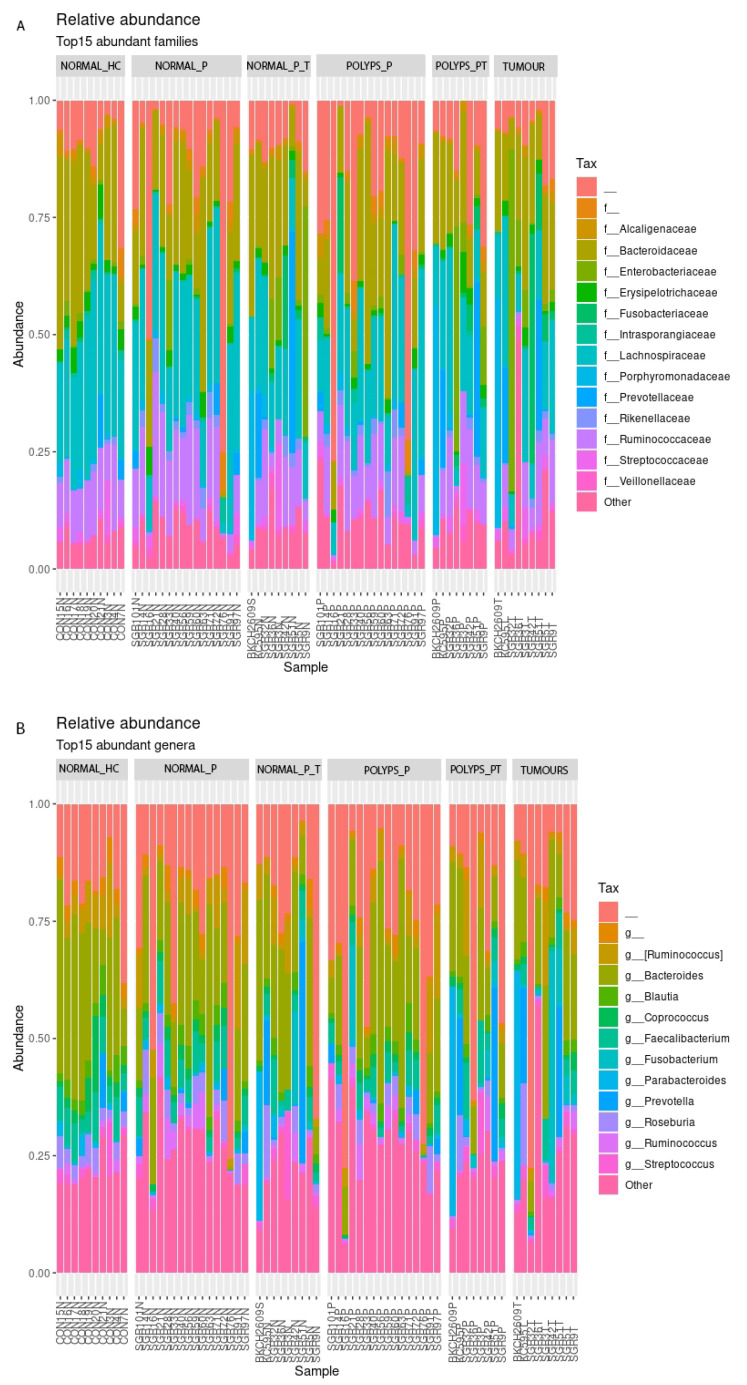
Relative abundance of most represented families (**A**) and genera within (**B**) sample groups. The symbol “_” indicates a group of unclassified sequences (after the taxonomic assignment procedure), while “f_” and “g_” indicate that a group of sequences belongs to an unannotated/unnamed families or genera.

The compositions of microbial communities among samples can be observed in Figure 1, in which the top 15 most represented (relative abundance) families and genera are shown.

We therefore implemented the QIIME2 ANCOM module to analyze sample microbial compositions and detect relevant variations among them. After the filtering step, feature tables of 108 × 68 (genus-collapsed features x samples) and 156 × 68 (species-collapsed features x samples) were used as input for the ANCOM analysis. At the genus level (L6), “*g__Fusobacterium*” (ANCOM W statistics = 39) showed a significant differential abundance in synchronous lesions as compared to healthy controls and adenomas (Supplementary File S1). At the species (L7) level, *Fusobacterium* of an unannotated species (W = 48) was found to significantly change among the synchronous lesions.

### 3.4. Discriminant Taxa from Pairwise Group Comparisons

Differential microbial abundance between group pairs was additionally investigated using the coda-lasso, clr-lasso and Selbal methods. Full results are given in Supplementary File S2.

We observed that six “genus-collapsed” taxa were found in common among the three methods in the HC cohort as compared to the synchronous samples: a feature “X” has been considered “common” when it is selected as “significantly differentially abundant” according to all techniques (although weights are calculated differently among methods). In the pairwise analysis “Normal_HC vs. Normal_P_T”, we pinpointed that *Staphylococcus* (custom id: 28) and *Actinobacillus* (id 101) showed greater abundance in healthy control colonic mucosa. In contrast, an unknown genus of the *Rikenellaceae* family (id 16) was more abundant in normal tissue of synchronous subjects. In the Normal_HC vs. Polyps_P_T analysis, a genus named *Gemmiger* (id 61) showed greater abundance in Normal_HC, while an unknown genus from the *Rikenellaceae* family (id 16) and one from Pseudomonas (id 105) were present at a greater abundance in specimens from Polyps_P_T. Of note, although no feature was in common among the three methods used for the Normal_HC vs. Tumor comparison, we noticed that a genus named *Fusobacterium* (id 83) showed greater abundance in tumor tissues as compared to HC under the coda-lasso, clr-lasso and ANCOM approaches.

Comparing the microbial composition from the HC cohort to the adenomas samples, we observed that two genera features were enriched in normal HC: *Staphylococcus* (id 28) vs. Normal_P and the Bifidobacterium vs. Polyps_P.

### 3.5. Differential Analysis of Inferred Microbial Pathways

A total of 1817 gene functions (defined by Enzyme Commission identifiers) and 370 metabolic pathways (defined by MetaCyc identifiers) have been inferred using the PICRUSt pipeline on the unfiltered QIIME2 feature table.

Using the PICRUSt “unstratified” (i.e., quantities are not stratified per sample) pathway count table, we identified differentially abundant pathways through pairwise group comparisons. Supplementary File S3 summarizes our comparative strategy together with the differentially abundant pathways retrieved by ALDEx2: the “ALDEx2_filtered” sheet shows a total of 11 differentially abundant items, although they emerge with the most relaxed “effect” cutoff, 1.

The pathways listed below appear to have a lower (mildly significant) predicted abundance in the Tumor group as compared to the normal control one: ectoine biosynthesis (MetaCyc identifier: P101-PWY), octane oxidation (P221-PW, myo-inositol degradation I (P562-PWY), catechol degradation to 2-hydroxypentadienoate II (PWY-5419), catechol degradation II (meta-cleavage pathway) (PWY-5420), mono-trans, poly-cis decaprenyl phosphate biosynthesis (PWY-6383), norspermidine biosynthesis (PWY-6562), methyl ketone biosynthesis (engineered) (PWY-7007), myo-, chiro- and scyllo-inositol degradation (PWY-7237), superpathway of L-alanine biosynthesis (PWY0-1061) and mycothiol biosynthesis (PWY1G-0). No differences appeared for the other four binary comparisons.

### 3.6. Fusobacterium Nucleatum Detection and Quantification

**Figure 2:**
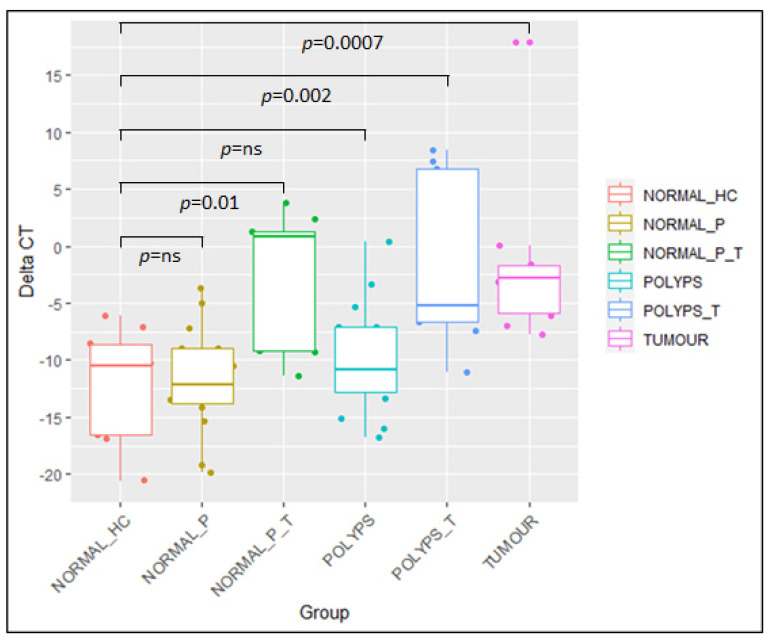
Boxplot for ∆CT values of *Fusobacterium nucleatum* expression within sample groups.

By means of RT-qPCR, we quantified the amount of *Fusobacterium nucleatum* by real-time qPCR in synchronous tissues of CRC patients, adenomas, and HC tissues. *Fusobacterium nucleatum* were shown to be enriched among synchronous tissues compared to HC; the differences were statistically significant in tissue biopsies pinched from Tumor (Wilcoxon-test *p*-value = 0.0007), Polyps_P_T (*p*-value = 0.002) and adjacent Normal_P_T tissues (*p*-value = 0.01) (Figure 2).

The amount of *Fusobacterium nucleatum* was almost similar to that of HC in both Polyps_P (*p*-value = 0.34) and from adjacent Normal_P tissues (*p*-value = 0.93).

## 4. Discussion

In recent decades, researchers have gathered evidence of a correlation between microbiota, specific strains, signaling pathways, microbiota-related metabolites, and the risk of colorectal cancer [33]. However, it is not completely understood whether a specific microbiota profile contributes to the development of sporadic CRC, if and which changes occur in the microbial composition during the progression of colorectal cancer, and if microbiota alterations in the precancerous condition (colorectal polyps) can predict progression toward a neoplasia.

In this study, we chose to study the mucosal microbiota, believing that it mostly interacts with neoplastic lesions and the immune system [34].

We performed targeted bacterial 16S rRNA gene sequencing and qPCR on bacterial DNA to examine the crosstalk between microbiota, polyps, and colorectal cancers, and to identify possible differences or gradients in concentration of the microbial composition of patients with adenomas alone and those with synchronous lesions.

Even though we identified differences in the abundance of a few species between synchronous CRC lesions and HC, a deep microbial dysbiosis was not observed, at least when using crude alpha and beta diversity indices. Under the alpha metrics, the microbial communities were characterized by a lower diversity, although a weak association was observed when Tumor samples were compared with HC ones. Under multiple hypothesis testing (*q*-values), though, the results were not significant.

Microbial compositions were different in terms of the beta diversity between Tumor specimens and HC, according to the Bray–Curtis dissimilarity, while all the comparisons gave significant results according to the Jaccard similarity index, independently from synchronous CRC specimens and from adenomas ones. Thus, the groups are qualitatively and significantly dissimilar from each other, as determined by calculating Jaccard distances. On the other hand, considering counts of shared features (with respect to the total feature number within a sample pair), only the Tumor group was found to significantly differ from the HC one.

The magnitude of these microbial alterations is in line with other studies elegantly reviewed by Costa and her collaborators [35], in which the mucosal microbiomes between CRC patients and healthy controls were analyzed.

We showed two major subsets of bacterial genera. The first subset showed a greater abundance in healthy controls, and the second was enriched in subjects with synchronous tumors and adenomas, while no taxa were enriched in patients with only adenomas, as highlighted by at least three feature selection methods. The first cluster represents bacteria associated with wellbeing, involved in intestinal homeostasis, which were all mostly abundant in HC: *Gemmiger, Actinobacillus*, *Staphylococcus* and *Bifidobacterium.*

The *Gemmiger* genus belongs to the Butyrate-producing genera, and was found to be decreased in the Polyps_P_T specimens. *Gemmiger* was also present at a decreased abundance in patients with blood in their stools in a study aimed at using the detection of blood in the patient’s stool as a marker of colon malignity [36].

The *Actinobacillus* genus was enriched in HC when compared to the Polyps_P_T cohort; to date, no data have been available in the literature on its involvement in CRC, except for its presence in normal mucosal swabs of CRC patients [37].

We identified the *Staphylococcus* genus as more abundant in HC when compared to both normal tissues from tumors (Normal_P_T) and adenomas. (Normal_P). *Staphylococcus* was found at a greater abundance in healthy tissues in a study reporting on the relationship between the tumor stage and microbiota composition [38].

Further strong negative associations have been shown related to the genera Bifidobacterium, identified in subjects with adenomas (Polyps_P), the results of which are in accordance with a study aimed to analyze the microbial composition at the mucosal level of human CRC [39] and at the fecal level in patients with early-stage colorectal cancer [40].

In the second subset, we have included those taxa that were more enriched in synchronous tissues, and which could be representative or stand as markers of the disease. *Pseudomonas* and an unknown genus from the *Rikenellaceae* family were more abundant in synchronous adenomas (Polyps_P_T), while a genus named *Fusobacterium* was identified in tumor tissues (Tumours).

The *Pseudomonas* genus was inversely correlated to CRC in several studies, according to which it exhibited a relatively higher abundance in normal tissues [39,41,42]. However, an increased relative abundance of this potential opportunistic pathogen was found in precancerous lesions [43].

Sun and colleagues [44] assessed a mouse colorectal cancer model, and speculated that the *Rikenellaceae* family functions as an opportunistic pathogen via the intensification of inflammation or the production of mutagenic toxins.

Overall, the most significant effect of our study was the identification of the *Fusobacterium* genus, present in the analyzed tools, as relatively more abundant in tumor specimens. Despite the small number of cases, the strength of our work is represented by the possibility of evaluating the effects of microbiota on healthy tissue, adenomas, and finally on the CRC in a homogeneous population with synchronous lesions.

Our analysis highlighted that *Fusobacterium nucleatum* was enriched not only in the tumor tissues, but more importantly, in both specimens with adenomas and the normal tissues of patients with synchronous lesions. Taken together, these results are consistent with those from emerging studies supporting the key role of *Fusobacterium nucleatum* in carcinogenesis.

*Fusobacterium nucleatum*, although it is a common type of oral microbiota with a symbiotic relationship with its hosts, has been frequently found to be enriched in patients with colorectal cancer [33]. In addition, there is evidence that the oral concentration of *Fusobacterium nucleatum* can influence colon tissue concentrations, and predict colon cancer prognosis [45]. *Fusobacterium nucleatum* has been discovered to impact cancer cells or modulate the tumor microenvironment, influencing the progression, metastasis, and chemoresistance of CRC by affecting cancer cells or regulating the tumor microenvironment [46].

This organism has been correlated with the DNA methylation of genes within the inflamed colonic mucosa by contributing to the progression of colorectal neoplasia [47], and has been shown to upregulate DNA methyltransferase, resulting in the aberrant epigenetic regulation of tumor-suppressor genes in colonic epithelial cells [48]. Moreover, the *Fusobacterium nucleatum* induces inflammation and alteration in the intestinal barrier of colorectal cancer tissue, and has been associated with shorter survival [49] and specific somatic mutated genes [50].

Recently, it has been shown that *Fusobacterium nucleatum* could modulate the tumor microenvironment and influence the response to chemo-radiotherapy in patients with advanced rectal cancer [51]. *Fusobacterium nucleatum* positivity after neoadjuvant chemo-radiotherapy significantly increased the risk of relapse. Of note, the disappearance of *Fusobacterium nucleatum* after this induction was correlated with a strong increase in CD8+ T cells. On the contrary, tumors showing persistence of *Fusobacterium nucleatum* after chemo-radiotherapy did not show an increase in CD8+ T cells in post-treatment samples compared with baseline [48].

At present, several studies and meta-analyses [52,53,54] have focused on *Fusobacterium nucleatum* as a compelling biomarker for CRC, although they have yielded no complete agreement regarding either the use of the matrices analyzed (stool vs. mucosal specimens) or the methodologies used (qPCR versus 16S rRNA gene amplicon). To date, hope is primarily placed on the use of droplet digital PCR (ddPCR) to identify and quantify low-abundance targets in the hope of accurately detecting potentially oncogenic bacteria, and evidence has also recently been reported related to the detection of the *Fusobacterium nucleatum* in FFPE CRC tissues [55].

Altogether, these studies show that CRC patients carry a high abundance of *Fusobacterium nucleatum,* and have clarified its role as a pro-carcinogenic bacterium in various stages of CRC, considering it a leading candidate as both an oncomicrobe and a driver of carcinogenesis.

Using qPCR, we analyzed all the samples by focusing on *Fusobacterium nucleatum.* Our data highlight the importance of this microorganism, as it was equally distributed in all the tissues pinched from synchronous lesions, but not in those from only adenomas. *Fusobacterium nucleatum* was significantly enriched not only in tumor tissues, but also in synchronous noncancerous adenomas and adjacent normal tissues. Otherwise, no significant differences have been found in patients with adenomas, both in polyps and normal tissues. Altogether, our 16S data, derived by means of an ensemble of metagenomics analysis tools and b quantitative real-time PCR, pinpointed the importance of *Fusobacterium nucleatum* as the most representative taxa involved in synchronous lesions, which is almost absent in those subjects with adenomas alone, suggesting its involvement in cancer genesis. Notably, these findings are consistent with those of earlier reports [7,56], implying that *Fusobacterium nucleatum* influences tumorigenesis and plays a key role in distant metastases [10].

From the analysis of the metabolic pathways obtained from the microbial gene content inferred from 16S rRNA gene data, we have shown that the microbial composition of the control group is enriched in bacteria, with the genetic ability to synthesize some “protective bioactive compounds” crucial for the wellbeing of the host, primarily ectoine and mycothiol. Ectoine is a natural powerful cytoprotectant compound found in higher concentrations in halophilic microorganisms, and acts as a compatible solute for the survival of osmotic stress [57], with protective effects on the epithelial barrier during inflammation [58]. Mycothiol is a potent antioxidant compound, and serves as a glutathione (GSH) analogue [59].

We acknowledge that the study has several limitations. The small number of patients with synchronous lesions and adenomas makes it more difficult to generalize the results that we have taken as hypothesis-generating, even if the number of enrolled patients is in line with that value for a monocentric study. Second, the lack of information regarding follow-ups and outcomes hinder the possibility of inferring a correlation. However, the applied computational strategy has enabled us to identify and quantify differences in microbial and functional content among synchronous lesions, adenomas, and control samples. In solving such limitations, we experienced numerous technical challenges. First, we obtained high-quality reads, which required the application of rarefaction tests and feature/sample filtering criteria to obtain reliable diversity metrics and remove ultra-rare taxa. We also took advantage of several (even alternative) bioinformatic packages that, while providing valuable results, also have their limitations. For example, functional differential abundance analyses were carried out on predicted gene quantities, and pathways were reconstructed through these quantities.

In this single-center study of the mucosal microbiota obtained from synchronous lesions, adenomas and healthy controls, weak microbial stability was found, mostly via a comparison among groups. The applied computational strategy identified a small number of both protective taxa and high-risk bacteria associated with neoplasia. If the first subset is enriched in bacteria and functional pathways that play a key role in the wellbeing of the host, the second is particularly enriched in *Fusobacterium nucleatum*, probably the most representative carcinogenic bacteria that will be of extensive interest.

Here, we have pinpointed that *Fusobacterium nucleatum* was enriched not only in the tumor tissues, but more importantly, in both specimens with adenomas and in normal tissues of patients with synchronous lesions, whilst subjects with only adenomas showed *Fusobacterium nucleatum* levels that were almost similar to those of the HC, suggesting equal distribution in all the synchronous lesions, as well as cancer-specificity. These data may shed new light on the role of *Fusobacterium nucleatum* in colorectal cancer. We hope that in future studies, a much larger population-scale study and more precise detection approaches (i.e., ultrasensitive droplet digital PCR techniques applied on several specimens including FFPE tissues) may solve this issue, and demonstrate the bacteria to be a leading candidate for use as both an oncomicrobe and a driver of carcinogenesis, as well as a comprehensive biomarker of CRC.

## Figures and Tables

**Table 1:** General characteristics of patients with CRC and synchronous polyps, adenomatous polyps, and healthy controls subjects.

Characteristics		Healthy Controls	Adenomatous Polyps	CRC and Synchronous Polyps
Individuals	total number	10	16	9
	ratio of females to males	5/5	3/13	2/7
Age, years	median	47	70	66
	range	27–66	41–82	51–89
Age of diagnosis	early (<50)	-	2	0
	late (>50)	-	14	9
Polyp localization	proximal to splenic flexure	-	5	3
	distal to splenic flexure	-	11	6
Cancer localization	proximal to splenic flexure	-	-	3
	distal to splenic flexure	-	-	6
Concordance localization of cancer and polyp	proximal to splenic flexure	-	-	6/6
	distal to splenic flexure	-	-	3/3
Polyp dysplasia	low grade	-	6	4
	high grade	-	10	5
Features of advanced adenoma (AA)	ratio of AA to total		14/16	6/9
TNM stage	I	-	-	0
	II	-	-	3
	III	-	-	4
	IV	-	-	1
	NA	-	-	1
Grading (WHO)	G1	-	-	2
	G2	-	-	6
	G3	-	-	0
	NA	-	-	1

**Table 2:** Summary of pairwise group comparisons for four alpha diversity indexes. Benjamini and Hochberg corrected *p*-values (*q*-values) for Kruskal–Wallis tests are shown.

	Healthy Colon				
	vs.				
	NORMAL_P_T	POLYPS_P_T	TUMOUR	NORMAL_P	POLYPS_P
Pielou’s evenness	0.478	0.478	0.236	0.833	0.486
Faith’s Phylogenetic Distance	0.723	0.615	0.932	0.616	0.723
Number of observed Features	0.472	0.382	0.490	0.382	0.472
Shannon’s Entropy	1	0.481	0.481	0.237	0.583

**Table 3:** Summary of pairwise group comparisons for beta diversity measures. Benjamini and Hochberg corrected *p*-values (*q*-values) for PERMANOVA tests are shown.

	Healthy Colon				
	vs.				
	NORMAL_P_T	POLYPS_P_T	TUMOR	NORMAL_P	POLYPS_P
Bray–Curtis dissimilarity	0.050	0.060	0.030	0.126	0.063
Jaccard similarity	0.039	0.015	0.020	0.026	0.015
Unweighted UniFrac dissimilarity	0.461	0.09	0.324	0.324	0.324
Weighted UniFrac dissimilarity	0.531	0.531	0.531	0.556	0.531

## Data Availability

The data presented in this study are available in Supplementary Files. Due to ethical limitations, further data will be available on request.

## Supplementary Materials

The following supporting information can be downloaded at: https://www.mdpi.com/article/10.3390/microorganisms11051147/s1, File S1: sequencing statistics, taxonomical composition (feature counts), diversity and ANCOM analyses by QIIME2 pipeline; File S2: microbial differential abundance analysis results derived by coda_lasso, clr_lasso and Selbal methods; File S3: pathway prediction and associated differential abundance measurements by Picrust2 and ALDEx2 packages.
