# Supplementary material for: Mucosal Microbiota from Colorectal Cancer, Adenoma and Normal Epithelium Reveals the Imprint of Fusobacterium nucleatum in Cancerogenesis
Source: Microorganisms. 2023 Apr 28;11(5):1147. doi: 10.3390/microorganisms11051147 (PMC10222013; doi:10.3390/microorganisms11051147)
Supplement: Supplementary file 1 [file microorganisms-11-01147-s001.zip › Supplementary_File_S1.pdf]

Supplementary File S1

1.1 – Sequencing and alignment statistics.

Per- sample Sequencing statistics. First column: sample identifier; columns 3 – 9: absolute counts and percentages for surviving reads after QIIME2 DADA2 implementation.

	Sample ID	# reads	# filtered	% filtered	#denoised	#merged	% input merged	# non-chimeric	% non-chimeric
Healthy Colon	CON15N	255365	196315	76.88	194364	189441	74.18	27407	10.73
Healthy Colon	CON16N	116845	87151	74.59	86202	83967	71.86	15175	12.99
Healthy Colon	CON17N	94682	73412	77.54	72109	70418	74.37	11228	11.86
Healthy Colon	CON18N	286955	232609	81.06	230427	224315	78.17	26480	9.23
Healthy Colon	CON19N	127674	98086	76.83	96600	93791	73.46	15828	12.4
Healthy Colon	CON20N	114042	89346	78.34	88109	85471	74.95	13478	11.82
Healthy Colon	CON21N	120686	93008	77.07	91530	89345	74.03	14947	12.39
Healthy Colon	CON3N	666215	530237	79.59	526459	513893	77.14	73116	10.97
Healthy Colon	CON4N	109006	86310	79.18	85081	82486	75.67	15519	14.24
Healthy Colon	CON7N	206676	136952	66.26	133993	126095	61.01	25752	12.46
NORMAL_P_T	KC595N	295298	221252	74.92	217162	209253	70.86	35621	12.06
POLYPS_P_T	KC595P	310855	225684	72.6	221723	215376	69.29	38200	12.29
TUMOUR	KC595T	339736	251721	74.09	249358	244514	71.97	39525	11.63
NORMAL_P_T	SGR32N	173104	126841	73.27	125545	122584	70.82	18825	10.87
POLYPS_P_T	SGR32P	233852	176488	75.47	174676	171087	73.16	32349	13.83
TUMOUR	SGR32T	292041	211250	72.34	209503	206406	70.68	33730	11.55
NORMAL_P_T	SGR36N	119399	92426	77.41	91228	89583	75.03	15819	13.25
POLYPS_P_T	SGR36P	181187	133057	73.44	131622	127628	70.44	19696	10.87
TUMOUR	SGR36T	129045	101017	78.28	100064	98426	76.27	15224	11.8
NORMAL_P_T	SGR3N	281413	206553	73.4	205474	202336	71.9	26555	9.44
POLYPS_P_T	SGR3P	252332	190365	75.44	187103	169797	67.29	11009	4.36
TUMOUR	SGR3T	287284	216347	75.31	215048	212095	73.83	34489	12.01
NORMAL_P_T	SGR42N	196342	143747	73.21	142057	139329	70.96	29479	15.01
POLYPS_P_T	SGR42P	125741	89191	70.93	87561	84892	67.51	20576	16.36
TUMOUR	SGR42T	175592	131771	75.04	130355	128139	72.98	29189	16.62
NORMAL_P_T	SGR51N	227932	169578	74.4	164633	144762	63.51	17303	7.59
POLYPS_P_T	SGR51P	157662	117602	74.59	116368	113569	72.03	18498	11.73
TUMOUR	SGR51T	231940	172213	74.25	169766	162511	70.07	27992	12.07
NORMAL_P_T	SGR9N	106047	76027	71.69	75104	72896	68.74	16103	15.18
POLYPS_P_T	SGR9P	181972	128814	70.79	125438	119759	65.81	29961	16.46
TUMOUR	SGR9T	304090	228408	75.11	226323	221408	72.81	41058	13.5
NORMAL_P_T	BKCH2609S	333033	260980	78.36	259758	256444	77	40465	12.15
POLYPS_P_T	BKCH2609P	74074	58378	78.81	57884	57325	77.39	7986	10.78
TUMOUR	BKCH2609T	83752	67451	80.54	66833	66111	78.94	9446	11.28
NORMAL_P_T	SGR5N	169509	130650	77.08	128945	125810	74.22	21692	12.8
TUMOUR	SGR5T	239993	182865	76.2	181629	177807	74.09	20138	8.39
NORMAL_P	SGR101N	160677	110082	68.51	107132	102547	63.82	17934	11.16
POLYPS_P	SGR101P	101458	74745	73.67	73879	72707	71.66	9103	8.97

NORMAL_P	SGR14N	379059	275612	72.71	273071	265959	70.16	40756	10.75
POLYPS_P	SGR14P	246283	178247	72.37	176493	172196	69.92	26873	10.91
NORMAL_P	SGR16N	172611	110517	64.03	109504	105615	61.19	13826	8.01
POLYPS_P	SGR16P	213100	147047	69	146068	144063	67.6	26695	12.53
NORMAL_P	SGR21N	245402	179500	73.15	175273	157602	64.22	22261	9.07
POLYPS_P	SGR21P	180618	133651	74	130843	119934	66.4	19524	10.81
NORMAL_P	SGR28N	232924	176568	75.8	174397	166780	71.6	29093	12.49
POLYPS_P	SGR28P	279993	201067	71.81	198720	193181	68.99	37715	13.47
NORMAL_P	SGR33N	422669	304567	72.06	301557	294558	69.69	47242	11.18
POLYPS_P	SGR33P	207413	150672	72.64	148167	141461	68.2	30381	14.65
NORMAL_P	SGR40N	339467	251277	74.02	247770	238874	70.37	42854	12.62
POLYPS_P	SGR40P	451013	332711	73.77	328626	316246	70.12	53974	11.97
NORMAL_P	SGR56N	204686	152330	74.42	149627	143366	70.04	28388	13.87
POLYPS_P	SGR56P	168271	133015	79.05	131974	129349	76.87	14374	8.54
NORMAL_P	SGR59N	172571	115817	67.11	114140	110392	63.97	22333	12.94
POLYPS_P	SGR59P	115292	78835	68.38	77306	74492	64.61	17465	15.15
NORMAL_P	SGR60N	188689	136461	72.32	134228	129151	68.45	27507	14.58
POLYPS_P	SGR60P	195044	148946	76.37	146770	142308	72.96	27685	14.19
NORMAL_P	SGR63N	157655	113353	71.9	111968	109040	69.16	22281	14.13
POLYPS_P	SGR63P	122765	85202	69.4	83918	80247	65.37	15379	12.53
NORMAL_P	SGR71N	433740	315684	72.78	312411	300678	69.32	48428	11.17
POLYPS_P	SGR71P	144497	104103	72.05	102634	100337	69.44	19513	13.5
NORMAL_P	SGR72N	89728	65383	72.87	63741	58482	65.18	11140	12.42
POLYPS_P	SGR72P	50463	37994	75.29	37269	35927	71.19	6417	12.72
NORMAL_P	SGR76N	405619	255149	62.9	250892	232364	57.29	58694	14.47
POLYPS_P	SGR76P	322303	210095	65.19	207468	194909	60.47	44731	13.88
NORMAL_P	SGR91N	120803	80193	66.38	77710	74076	61.32	14091	11.66
POLYPS_P	SGR91P	231825	161900	69.84	158702	152691	65.86	30239	13.04
NORMAL_P	SGR97N	85123	61249	71.95	59952	56921	66.87	10203	11.99
POLYPS_P	SGR97P	141284	110165	77.97	109178	107205	75.88	11576	8.19

1.2 - Feature distribution per sample ("per sample" column) and global feature distribution ("per feature").

The number of high-quality sequence count ranges from 6417 to 73116, while feature abundance ranges from 1 to 30456 counts.

	Per sample	*Per feature*
Min. frequency	6417	1
1st quartile	15484	7
Median frequency	22307	16
3rd quartile	30873	44
Max. frequency	73116	30456
Mean frequency	25566	169

1.3 - Alpha diversity analysis.

We investigated Alpha diversity by calculating four different measures: Pielou's evenness (1.4.1), Faith's Phylogenetic Distance (1.4.2), Number of Observed Features (1.4.3) and Shannon's entropy (1.4.4). For each index, the following outcomes are provided: boxplot for group-specific index distribution (x-axis: "NORMAL_HC", "NORMAL_P", "NORMAL_P_T", "POLYPS_P", "POLYPS_P_T" and "TUMOUR"; y-axis: alpha-diversity index); Kruskal-Wallis test results (H statistics; p-value) for the global comparison of group-specific distribution; Kruskal-Wallis test results for pairwise group comparisons. Boxplot 1.4.4 is also reported in the main text, Figure 1. Analyses were performed through the "QIIME2 Diversity" module, using "**9103**" as cut-off for reads sampling depth (see main text for further details).

Pielou's evenness

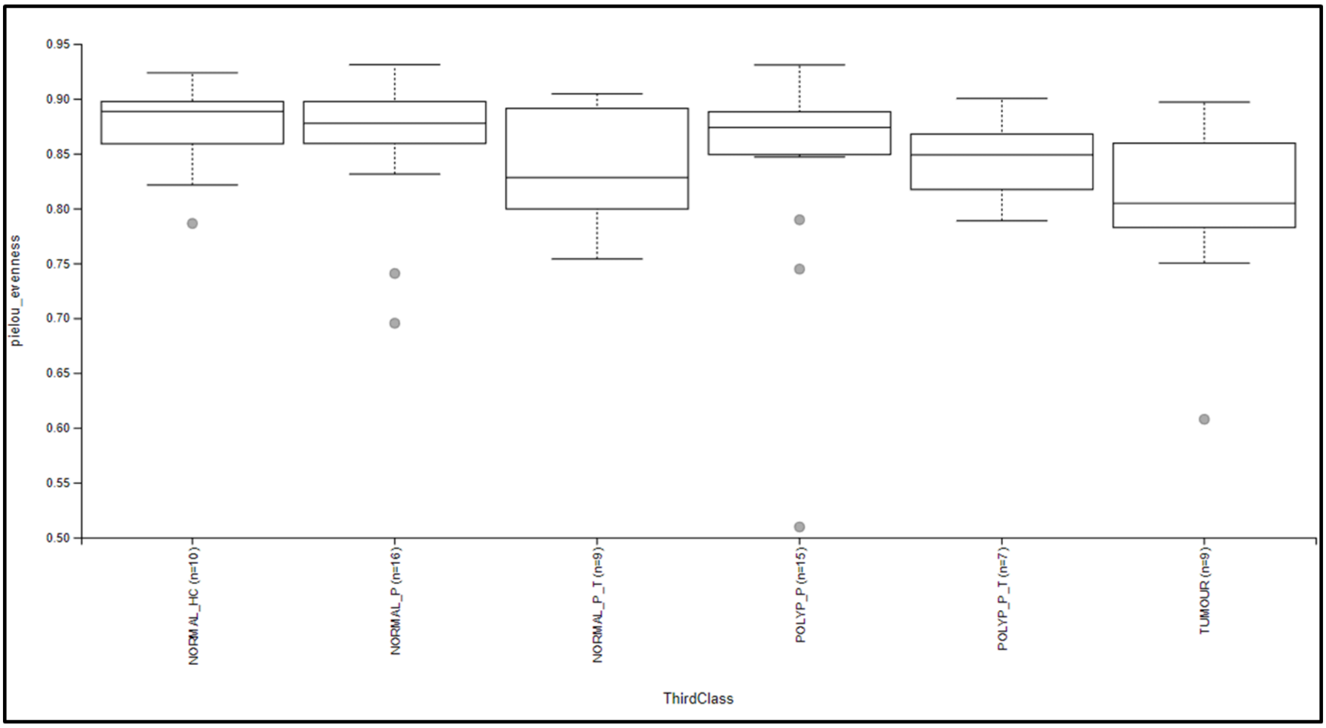

Kruskal-Wallis test (pairwise):

Group 1	Group 2	H	p-value	q-value
**NORMAL_HC (n=10)**	NORMAL_P (n=16)	0.044	0.833	0.833
	NORMAL_P_T (n=9)	1.706	0.191	0.478
	POLYP_P (n=15)	1.111	0.292	0.486
	POLYP_P_T (n=7)	1.867	0.172	0.478
	TUMOUR (n=9)	5.227	0.022	0.236
**NORMAL_P (n=16)**	NORMAL_P_T (n=9)	1.157	0.282	0.486
	POLYP_P (n=15)	0.264	0.607	0.700
	POLYP_P_T (n=7)	1.969	0.161	0.478
	TUMOUR (n=9)	4.628	0.031	0.226
**NORMAL_P_T (n=9)**	POLYP_P (n=15)	0.392	0.531	0.664
	POLYP_P_T (n=7)	0.070	0.791	0.833
	TUMOUR (n=9)	0.703	0.401	0.547
**POLYP_P (n=15)**	POLYP_P_T (n=7)	0.906	0.341	0.512
	TUMOUR (n=9)	2.689	0.101	0.478
**POLYP_P_T (n=7)**	TUMOUR (n=9)	1.482	0.223	0.478

Faith's Phylogenetic Distance

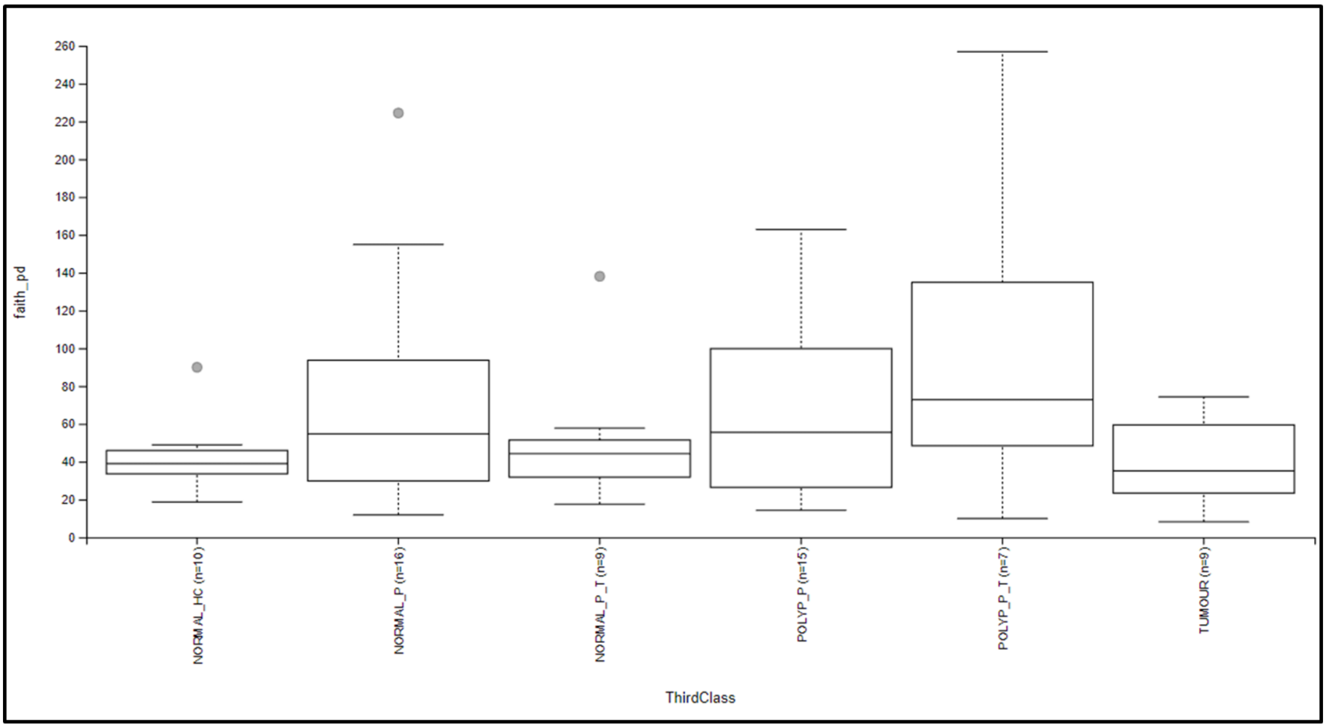

Kruskal-Wallis test (pairwise):

Group 1	Group 2	H	p-value	q-value
**NORMAL_HC (n=10)**	NORMAL_P (n=16)	1.344	0.246	0.616
	NORMAL_P_T (n=9)	0.240	0.624	0.724
	POLYP_P (n=15)	0.889	0.346	0.724
	POLYP_P_T (n=7)	1.609	0.205	0.616
	TUMOUR (n=9)	0.027	0.870	0.932
**NORMAL_P (n=16)**	NORMAL_P_T (n=9)	0.727	0.395	0.723
	POLYP_P (n=15)	0.006	0.937	0.937
	POLYP_P_T (n=7)	0.446	0.504	0.724
	TUMOUR (n=9)	1.846	0.174	0.616
**NORMAL_P_T (n=9)**	POLYP_P (n=15)	0.470	0.493	0.724
	POLYP_P_T (n=7)	1.751	0.186	0.616
	TUMOUR (n=9)	0.236	0.627	0.724
**POLYP_P (n=15)**	POLYP_P_T (n=7)	0.359	0.549	0.724
	TUMOUR (n=9)	1.352	0.245	0.616
**POLYP_P_T (n=7)**	TUMOUR (n=9)	2.692	0.101	0.616

Number of Observed Features

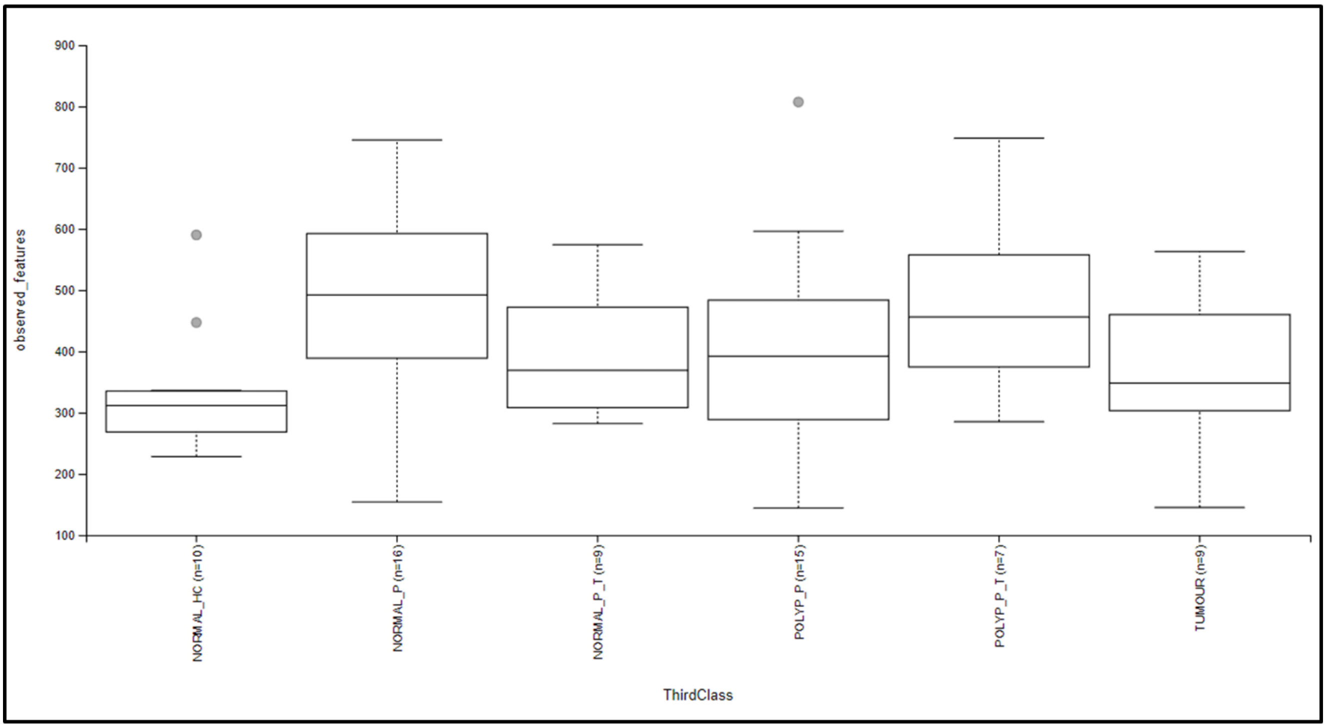

Kruskal-Wallis test (pairwise):

Group 1	Group 2	H	p-value	q-value
**NORMAL_HC (n=10)**	NORMAL_P (n=16)	4.225	0.040	0.382
	NORMAL_P_T (n=9)	1.707	0.191	0.472
	POLYP_P (n=15)	1.628	0.202	0.472
	POLYP_P_T (n=7)	3.810	0.051	0.382
	TUMOUR (n=9)	0.960	0.327	0.491
**NORMAL_P (n=16)**	NORMAL_P_T (n=9)	2.004	0.157	0.472
	POLYP_P (n=15)	1.502	0.220	0.472
	POLYP_P_T (n=7)	0.001	0.973	0.972
	TUMOUR (n=9)	2.696	0.101	0.472
**NORMAL_P_T (n=9)**	POLYP_P (n=15)	0.150	0.700	0.748
	POLYP_P_T (n=7)	1.011	0.315	0.491
	TUMOUR (n=9)	0.158	0.691	0.748
**POLYP_P (n=15)**	POLYP_P_T (n=7)	0.548	0.459	0.616
	TUMOUR (n=9)	0.470	0.493	0.616
**POLYP_P_T (n=7)**	TUMOUR (n=9)	1.236	0.266	0.491

Shannon entropy

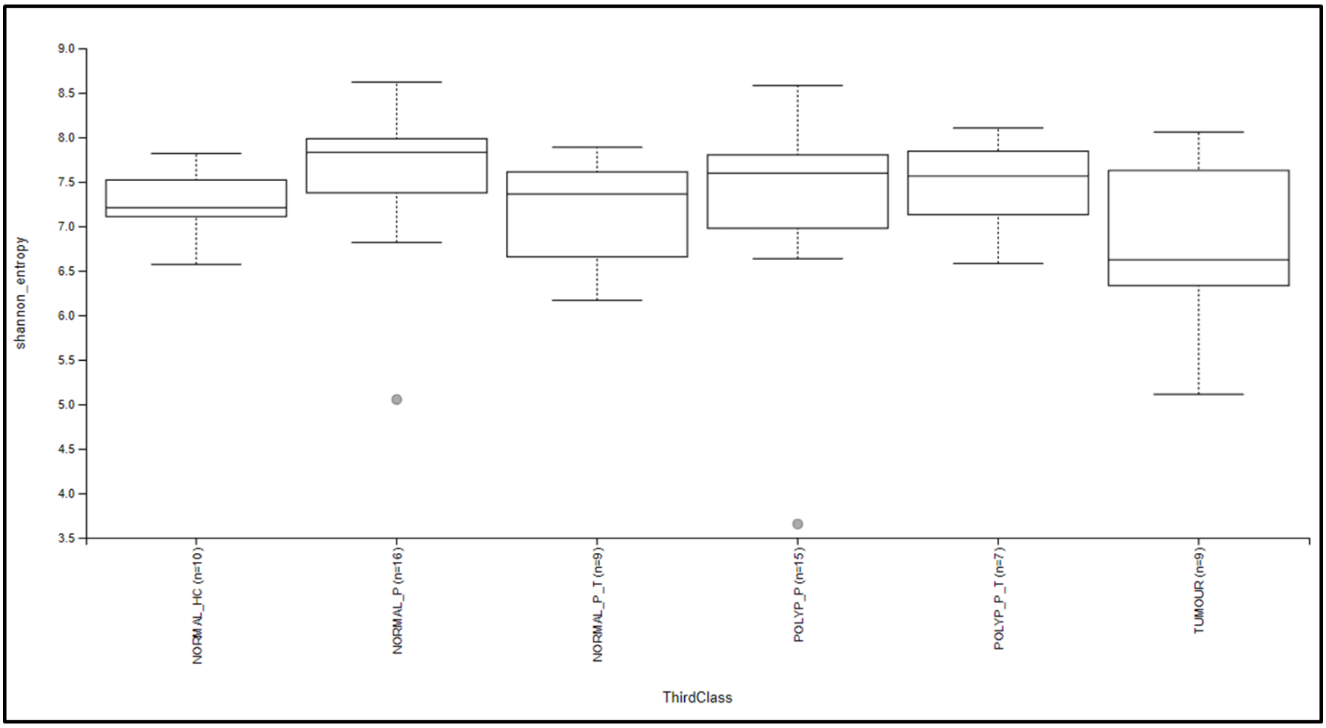

Kruskal-Wallis test (pairwise):

Group 1	Group 2	H	p-value	q-value
**NORMAL_HC (n=10)**	NORMAL_P (n=16)	4.011	0.045	0.239
	NORMAL_P_T (n=9)	0.000	1.0	1.0
	POLYP_P (n=15)	0.443	0.506	0.583
	POLYP_P_T (n=7)	1.373	0.241	0.481
	TUMOUR (n=9)	1.127	0.288	0.481
**NORMAL_P (n=16)**	NORMAL_P_T (n=9)	3.926	0.047	0.238
	POLYP_P (n=15)	1.314	0.252	0.481
	POLYP_P_T (n=7)	0.643	0.422	0.570
	TUMOUR (n=9)	4.388	0.036	0.238
**NORMAL_P_T (n=9)**	POLYP_P (n=15)	0.556	0.456	0.570
	POLYP_P_T (n=7)	1.235	0.266	0.481
	TUMOUR (n=9)	0.704	0.402	0.570
**POLYP_P (n=15)**	POLYP_P_T (n=7)	0.011	0.916	0.981
	TUMOUR (n=9)	1.963	0.161	0.481
**POLYP_P_T (n=7)**	TUMOUR (n=9)	2.042	0.153	0.481

1.4 - Beta diversity analysis.

Diversity between the two groups has been evaluated by considering four popular dissimilarity/distance methods: Bray-Curtis (1.5.1), Jaccard (1.5.2), Unweighted and Weighted Unifrac (1.5.3 and 1.5.4). For each method, the following outcomes are provided: a screenshot of three-dimensional Principal Coordinate Analysis ("3D-PCoA") plot; a table summarizing PERMANOVA test results for pairwise group comparisons. PCoA plots have been obtained by using the Emperor web-application within QIIME2 website (https://view.qiime2.org/); red dots indicate "NORMAL_HC" samples, blue "NORMAL_P", orange "NORMAL_P_T", green "POLYPS_P", purple "POLYPS_P_T" and yellow "TUMOUR". Furthermore, each table show details on comparing groups and their size, number of permutations, pseudo-F statistics, p-values and Benjamini-Hochberg adjusted p-value. PERMANOVA verifies the hypothesis that distances among samples with one class group differ from distances of these samples from samples of other groups. PCoA plot obtained by using Unweighted Unifrac distance is also reported in Main Text, as Figure 2.

Bray-Curtis

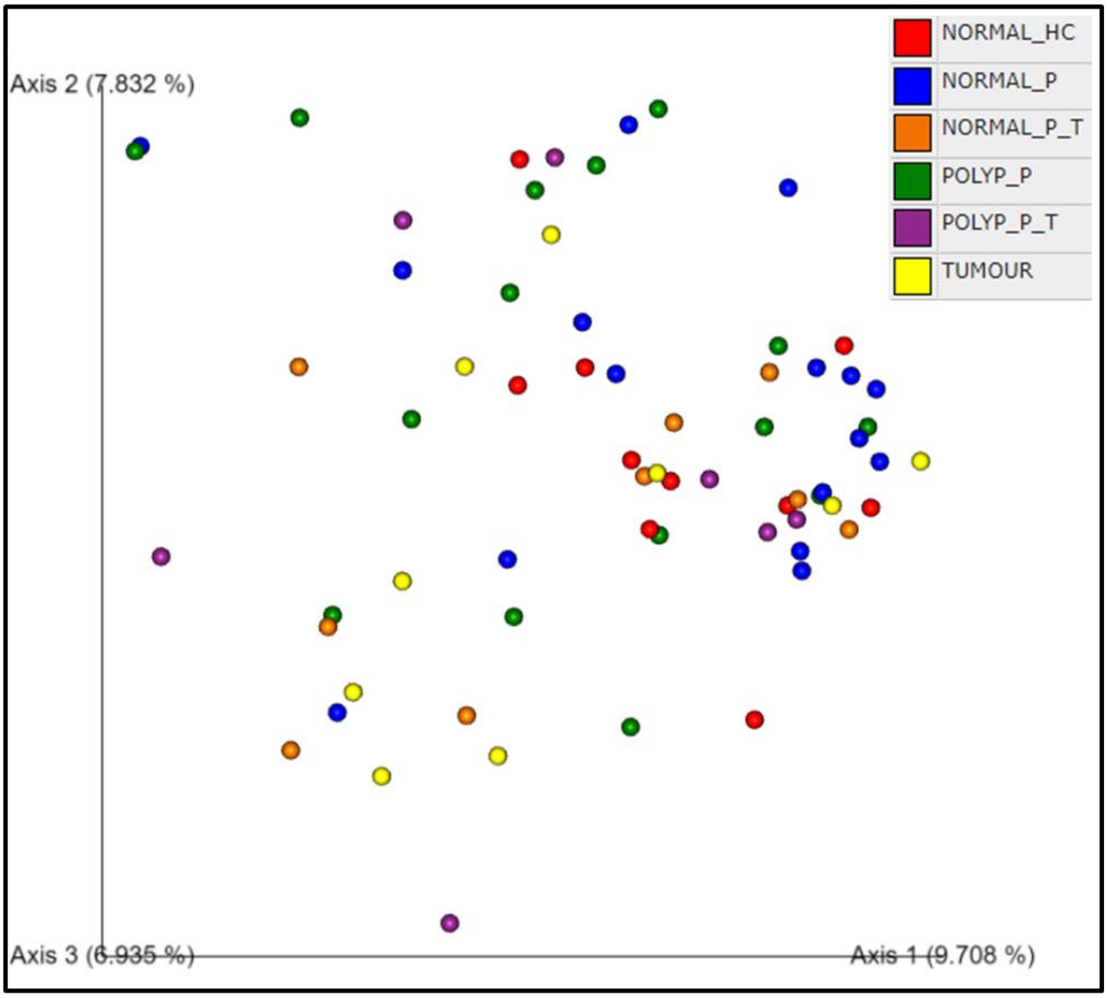

Group 1	Group 2	Sample size	Permutations	pseudo-F	p-value	q-value
**NORMAL_HC**	NORMAL_P	26	999	1.280	0.084	0.126
	NORMAL_P_T	19	999	1.468	0.009	0.050
	POLYP_P	25	999	1.468	0.025	0.063
	POLYP_P_T	17	999	1.441	0.020	0.060
	TUMOUR	19	999	1.642	0.002	0.030
**NORMAL_P**	NORMAL_P_T	25	999	1.463	0.016	0.060
	POLYP_P	31	999	0.578	0.968	0.999
	POLYP_P_T	23	999	1.303	0.083	0.126
	TUMOUR	25	999	1.583	0.010	0.050
**NORMAL_P_T**	POLYP_P	24	999	1.268	0.059	0.111
	POLYP_P_T	16	999	0.359	0.999	0.999
	TUMOUR	18	999	0.349	0.999	0.999
**POLYP_P**	POLYP_P_T	22	999	1.033	0.354	0.483
	TUMOUR	24	999	1.361	0.033	0.071
**POLYP_P_T**	TUMOUR	16	999	0.407	0.997	0.999

Jaccard

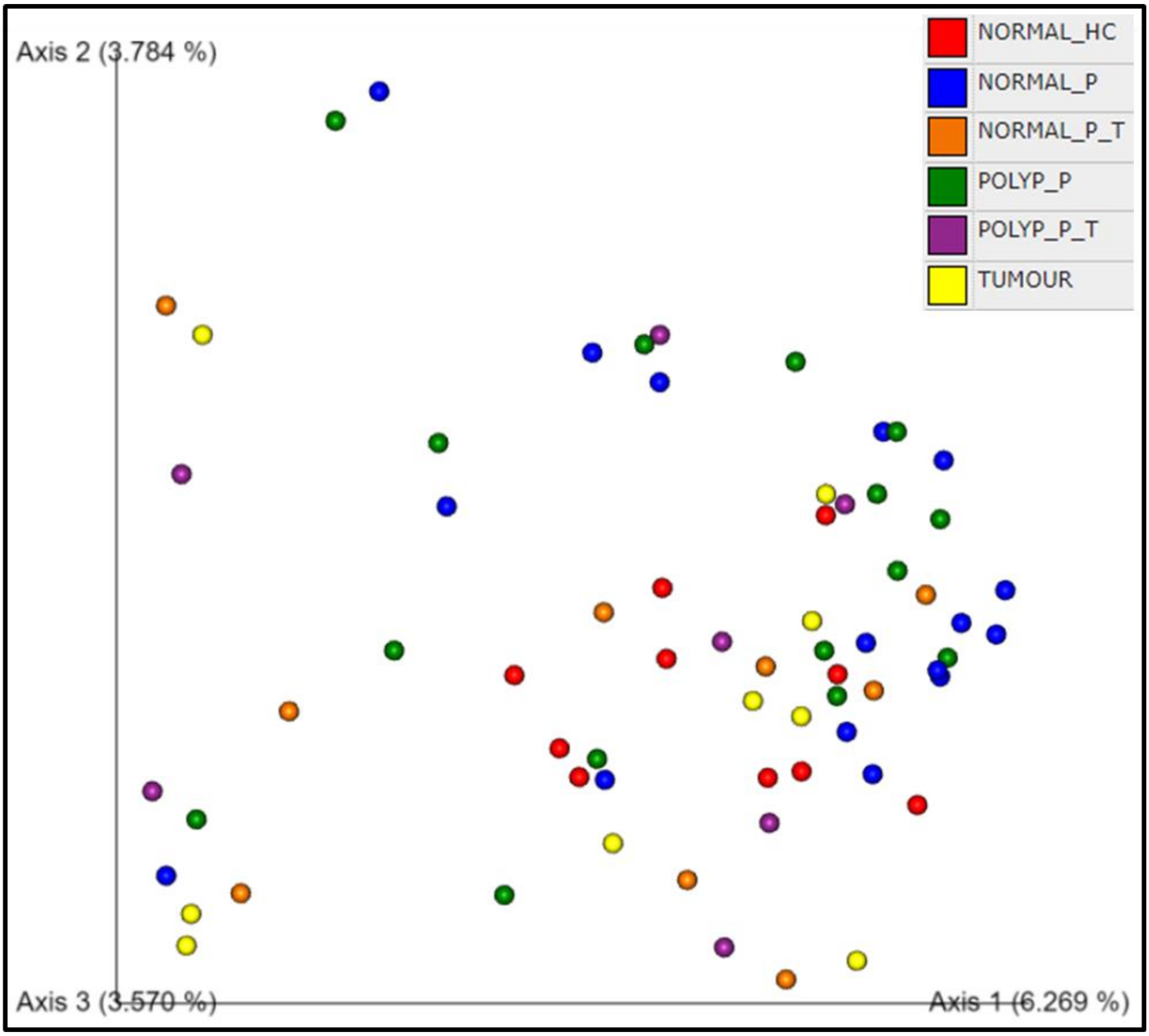

Group 1	Group 2	Sample size	Permutations	pseudo-F	p-value	q-value
**NORMAL_HC**	NORMAL_P	26	999	1.269	0.007	0.026
	NORMAL_P_T	19	999	1.224	0.013	0.039
	POLYP_P	25	999	1.267	0.002	0.015
	POLYP_P_T	17	999	1.343	0.001	0.015
	TUMOUR	19	999	1.280	0.004	0.020
**NORMAL_P**	NORMAL_P_T	25	999	1.188	0.043	0.081
	POLYP_P	31	999	0.670	0.997	1.0
	POLYP_P_T	23	999	1.205	0.033	0.077
	TUMOUR	25	999	1.222	0.036	0.077
**NORMAL_P_T**	POLYP_P	24	999	0.974	0.564	0.769
	POLYP_P_T	16	999	0.663	0.990	1.0
	TUMOUR	18	999	0.555	1.0	1.0
**POLYP_P**	POLYP_P_T	22	999	1.044	0.217	0.311
	TUMOUR	24	999	1.060	0.194	0.311

**POLYP_P_T**	TUMOUR	16	999	0.693	0.991	1.0

Unweighted Unifrac

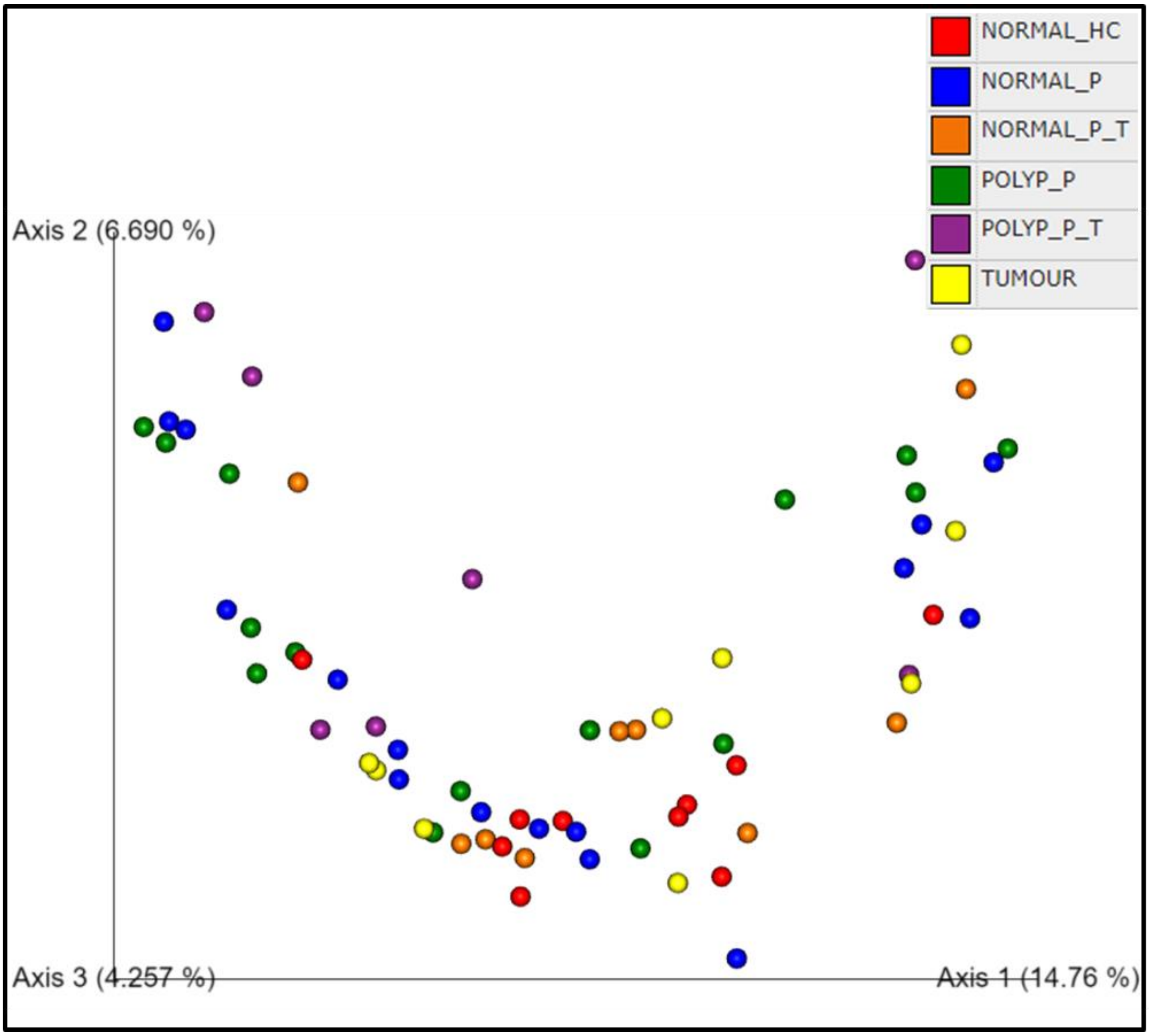

Group 1	Group 2	Sample size	Permutations	pseudo-F	p-value	q-value
**NORMAL_HC**	NORMAL_P	26	999	1.288	0.113	0.324
	NORMAL_P_T	19	999	1.041	0.369	0.464
	POLYP_P	25	999	1.436	0.051	0.324
	POLYP_P_T	17	999	1.651	0.006	0.090
	TUMOUR	19	999	1.249	0.076	0.324
**NORMAL_P**	NORMAL_P_T	25	999	1.170	0.203	0.324
	POLYP_P	31	999	0.709	0.967	0.967
	POLYP_P_T	23	999	1.115	0.216	0.324
	TUMOUR	25	999	1.179	0.161	0.324
**NORMAL_P_T**	POLYP_P	24	999	1.190	0.148	0.324
	POLYP_P_T	16	999	1.050	0.346	0.461
	TUMOUR	18	999	0.772	0.920	0.967
**POLYP_P**	POLYP_P_T	22	999	0.969	0.409	0.472

	TUMOUR	24	999	1.136	0.209	0.324
**POLYP_P_T**	TUMOUR	16	999	1.227	0.149	0.324

Weighted Unifrac

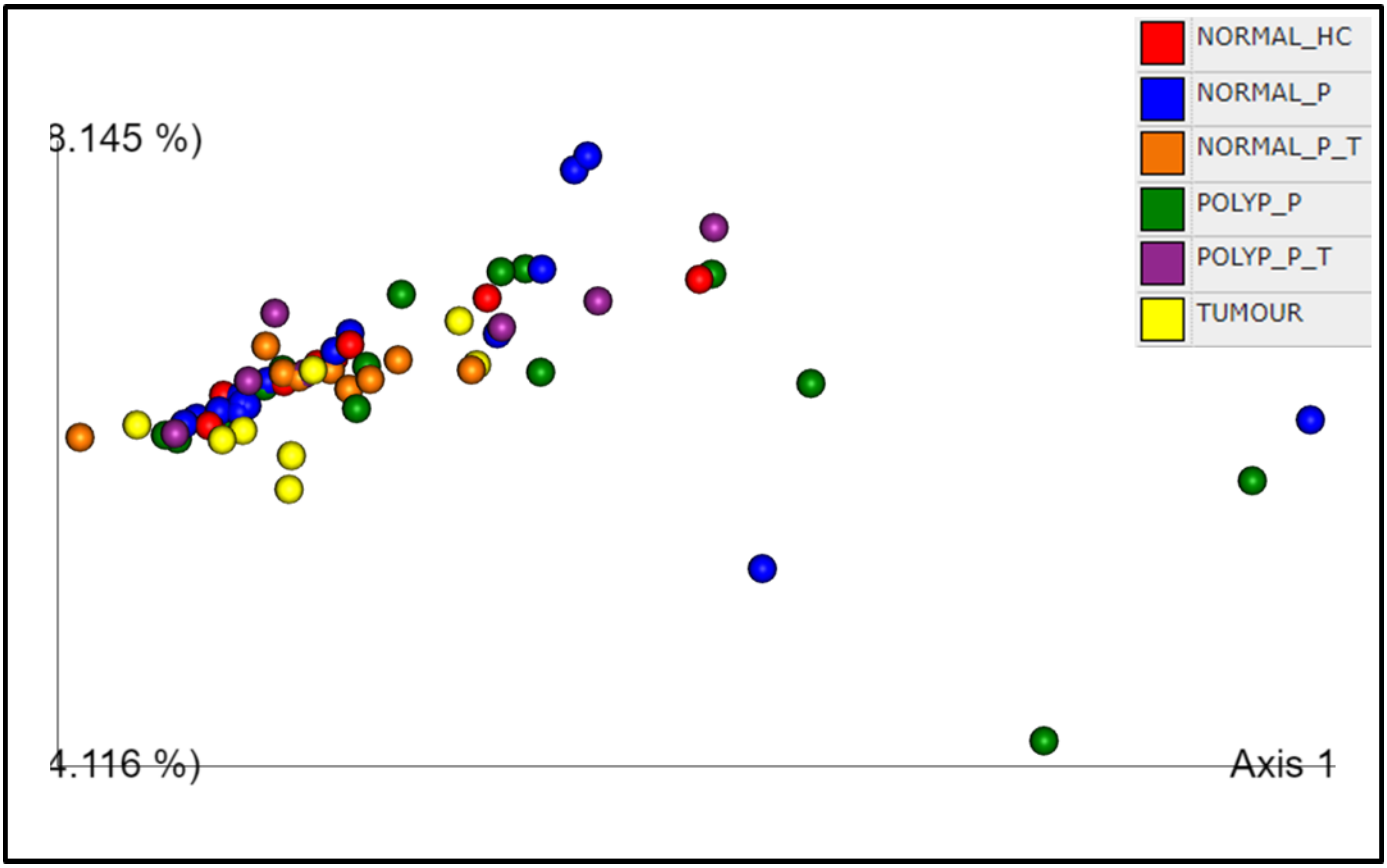

Group 1	Group 2	Sample size	Permutations	pseudo-F	p-value	q-value
**NORMAL_HC**	NORMAL_P	26	999	0.827	0.445	0.556
	NORMAL_P_T	19	999	0.912	0.390	0.532
	POLYP_P	25	999	2.131	0.126	0.532
	POLYP_P_T	17	999	1.079	0.326	0.532
	TUMOUR	19	999	1.294	0.262	0.532
**NORMAL_P**	NORMAL_P_T	25	999	1.411	0.212	0.532
	POLYP_P	31	999	0.595	0.519	0.560
	POLYP_P_T	23	999	0.593	0.581	0.581
	TUMOUR	25	999	1.693	0.185	0.532
**NORMAL_P_T**	POLYP_P	24	999	2.473	0.079	0.532
	POLYP_P_T	16	999	1.024	0.362	0.532
	TUMOUR	18	999	0.774	0.523	0.560
**POLYP_P**	POLYP_P_T	22	999	0.864	0.384	0.532
	TUMOUR	24	999	2.897	0.055	0.532
**POLYP_P_T**	TUMOUR	16	999	1.372	0.216	0.532

1.5 – ANCOM

QIIME2 ANCOM module was used to infer microbial genera or species that are differentially abundant across sample groups. Rare features and mitochondrial/plastid sequences were removed from the feature table (see Methods). Thus, features were collapsed according to QIIME2 taxonomical classification (based on GreenGenes resource) into "genera-collapsed" ("level 6") and "species-collapsed" ("level 7") features, for a total of 108 and 156 taxa, respectively. ANCOM Volcano Plot and statistical results table are provided.

Significant features are placed on the top-right corner of the Volcano Plot; the first table shows significant features together with the corresponding W statistics, i.e., the number of sub-hypotheses that have passed for a certain feature (ANCOM compares pairs of feature relative abundances). The second table shows the percentile abundance of relevant features across the sample groups. For example, a value of 100 at 50% percentile indicates that the detected feature has a maximum sequence count of 100 in the 50% of samples of an investigated group.

1.5.1 - Results for genus-collapsed features

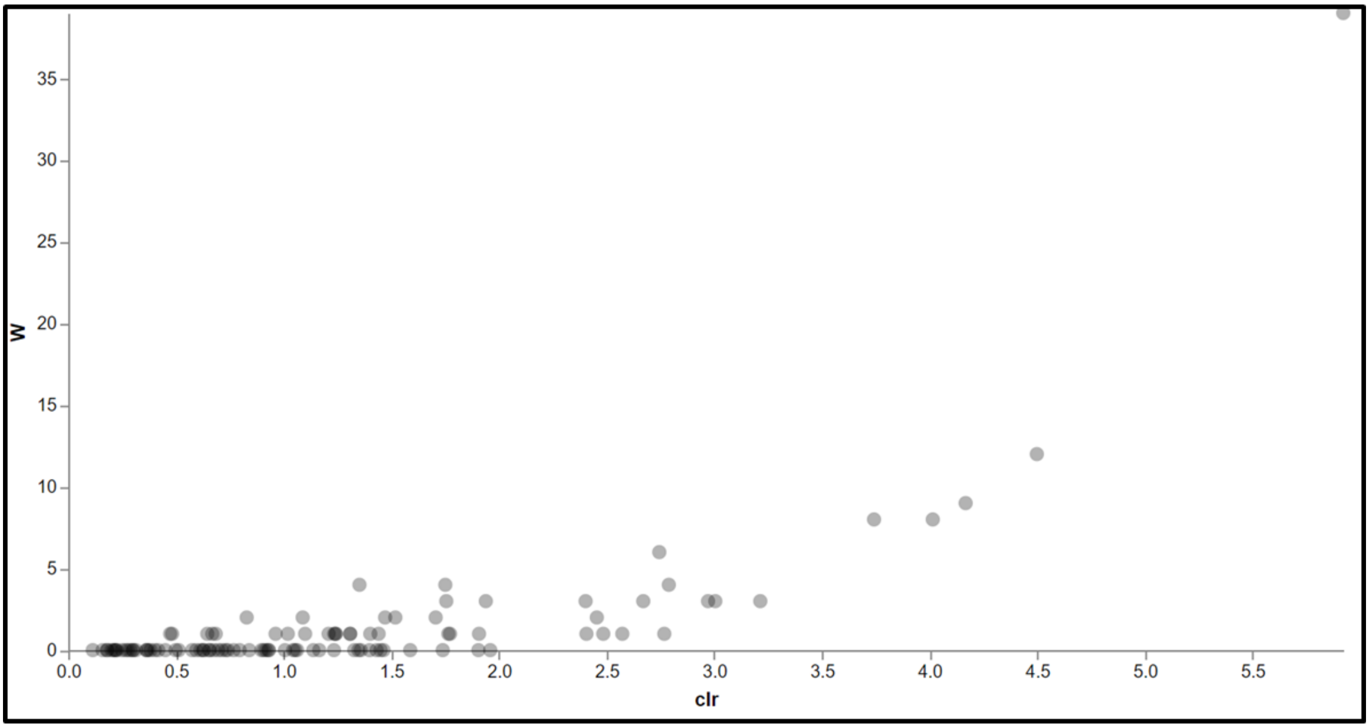

Feature	W
k__Bacteria;p__Fusobacteria;c__Fusobacteriia;o__Fusobacteriales;f__Fusobacteriaceae;g__Fusobacterium	39

Feature	Percentile	NORMAL_HC	NORMAL_P_T	POLYP_P_T	TUMOUR	NORMAL_P	POLYP_P
k__Bacteria;p__Fusobacteria;c__Fusobacteriia; o__Fusobacteriales;f__Fusobacteriaceae; g__Fusobacterium;	0.0	1.0	1.0	1.0	1.0	1.0	1.0
	25.0	1.0	40.0	66.5	67.0	1.0	1.0
	50.0	1.0	113.0	245.5	191.0	1.0	1.0
	75.0	77.75	342.0	427.0	907.0	19.25	55.75
	100.0	309.0	4836.0	881.0	12839.0	94.0	3018.0

1.5.2 - Results for species-collapsed features

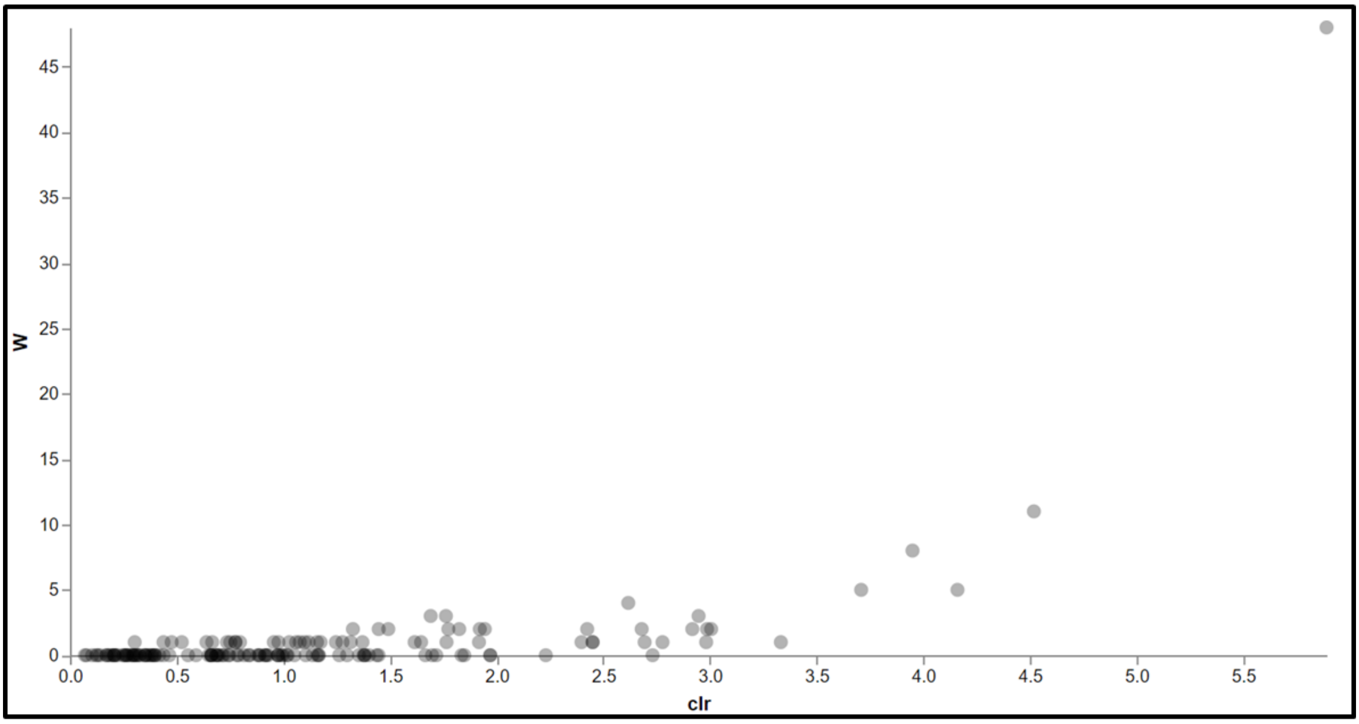

Feature	W
k__Bacteria;p__Fusobacteria;c__Fusobacteriia;o__Fusobacteriales;f__Fusobacteriaceae;g__Fusobacterium;**s__**	48

Feature	Percentile	NORMAL_HC	NORMAL_P	POLYP_P	NORMAL_P_T	POLYP_P_T	TUMOUR
k__Bacteria;p__Fusobacteria;c__Fusobacteriia; o__Fusobacteriales;f__Fusobacteriaceae; g__Fusobacterium;s__	0.0	1.0	1.0	1.0	1.0	1.0	1.0
	25.0	1.0	1.0	1.0	40.0	66.5	67.0
	50.0	1.0	1.0	1.0	113.0	245.5	191.0
	75.0	77.75	19.25	55.75	342.0	427.0	907.0
	100.0	309.0	94.0	3018.0	4836.0	881.0	12839.0
